# Research on the Interfacial Properties of AlSb Thin Films with Air Molecules

**DOI:** 10.3390/nano16171070

**Published:** 2026-08-27

**Authors:** Yang Wang, Ping Zhou, Xin Deng, Fujie Cai, Hanbing Ren, Weize Jiang, Fan Zhao, Huijin Song, Qiang Yan, Yingge Zhang

**Affiliations:** 1 Sichuan Rangtang Huadian New Energy Co., Ltd., Chengdu 624300, China; wangyang9@chd.com.cn (Y.W.); zhouping1234560824@163.com (P.Z.); 18828043373@163.com (X.D.); 18582196876@163.com (F.C.); 18090715636@163.com (W.J.); 2 School of Mechanical Engineering, Sichuan University, Chengdu 610106, China; renhanbing@scu.edu.cn; 3 College of Mechanical Engineering, Chengdu University, Chengdu 610106, China; zf1798966499@163.com (F.Z.); shj1437@163.com (Q.Y.); zhangyingge@cdu.edu.cn (Y.Z.)

**Keywords:** AlSb thin film, deliquescence, interface, molecular dynamics

## Abstract

AlSb film has attracted attention for its excellent properties, and many preparation methods have been explored. Herein, AlSb thin films were prepared by the DC magnetron co-sputtering method, and the interfacial behavior between the films and air molecules was investigated by X-ray diffraction (XRD), Auger electron spectroscopy (AES) testing, and density functional theory (DFT) calculations to elucidate the deliquescence process of AlSb thin films and its underlying mechanism. The results revealed that AlSb thin films exhibited Sb_2_O_4_ and Sb_2_O_5_ phases, while the thin films doped Cu no longer showed any Sb oxide phases after the film was exposed to air for one day. The chemical state of aluminum in the film remained stable along the depth direction, whereas antimony exhibited a pronounced gradient in chemical state from the surface to the interior. The oxidation state of Sb ions varied from −3 in the interior to +5 at the surface. The interaction between the (111) crystal plane of the AlSb film and air molecules is an exothermic process, with water molecules exhibiting the highest adsorption energy on the film surface, followed by oxygen molecules. The adsorption energies for nitrogen and carbon dioxide molecules were the lowest. Consequently, AlSb molecules readily combine with H_2_O molecules. Furthermore, doping the AlSb film with copper or zinc atoms effectively reduced the adsorption energy for water and oxygen molecules, offering a new approach to suppress the deliquescence and oxidation of AlSb thin films. This study provides an important theoretical foundation for subsequent research on this material system.

## 1. Introduction

The indirect band gap of AlSb is 1.6 eV, and the direct band gap is 2.22 eV, which is very suitable for the broad spectrum of visible light [1]; the theoretical conversion rate is high, up to 27% [2,3,4]. AlSb films show a high electron mobility of 200 cm^2^·V^−1^·s^−1^ and hole mobility of 400 cm^2^·V^−1^·s^−1^ [5]. It is widely used in field spintronics, in electro-optic and electronic devices [6], also in high-energy solar cells, P–N junction diodes and transistors [7,8], and other fields. This compound is used as a high-temperature semiconducting material [9], as an anode candidate for sodium/lithium-ion batteries [10,11], and is also promising as a photon detector [12]. Importantly, Al and Sb elements are abundant in the Earth’s crust and will not produce additional harmful substances during use, which makes it an environmentally friendly material [13]. Therefore, AlSb thin films will receive a lot of attention in the current situation of being unable to cope with traditional fossil energy, and it also determines that they will have huge development potential.

AlSb thin films have been prepared by magnetron sputtering an alloy target or a geometric target, or an Al, Sb target [2,4], co-evaporation [14], pulsed laser deposition [15], and electrodeposition [16,17], respectively. Meanwhile, the structure, Electrical properties, and Optical properties have been researched. Compared with GaAs films, the faster hydrolysis rate of AlSb film in aqueous medium affects its performance in solar cells, especially under short-circuit current [16,18]. Sattar and Farhan deposited AlSb: Zn thin films by the chemical bath method, and their results show that these electrodeposited films show unique development potential when applied to photonics, photocatalysis, and the energy industry and other fields [19]. Therefore, we need to solve the deliquescence problem of AlSb films and find ways to improve the stability of AlSb films. Mingqiang Li proposed a sandwich structure of Ni/AlSb/acrylic resin/Ni multilayer films to prevent AlSb films from contacting the atmosphere and thus prevent AlSb films from deliquescence [20]. Yixuan Pei used AlSb/AlSb: Cu composite films to study the properties and the inhibition of deliquescence of AlSb films [21]. Jianxiong He measured the deliquescence process of AlSb thin films by experimental observation [3]. However, the deliquescent mechanism and theoretical verification of AlSb films have not been reported on in detail.

In this paper, AlSb thin films were prepared by co-sputtering of Al and Sb in a magnetron sputtering system; the interfacial properties of AlSb films with air molecules were investigated by AES and Density functional analysis (DFT) and Molecular dynamics.

## 2. Experiment

### 2.1. Preparation Process and Testing Conditions

AlSb films were deposited on quartz glass at room temperature via direct-current (DC) magnetron sputtering in a vacuum chamber with a base pressure of 5 × 10^−3^ Pa and annealed at 400 °C with a holding time of 60 min in vacuum conditions. The AlSb films were prepared at 0.75 A/430 V power and 0.35 Pa pressure for 30 min.

The X-ray diffraction (XRD) measurement was performed using a Rigaku DMAD-RCDX-1000 diffractometer using Cu Ka radiation from Rigaku Corporation (Tokyo, Japan). The Auger electron spectroscopy (AES) of AlSb thin films was conducted at the State Key Laboratory of the Institute of Engineering Physics, Chinese Academy of Sciences, using a PHI-600 Auger electron spectrometer from ULVAC-PHI Corporation (Tokyo, Japan).

### 2.2. Physical Analysis and Chemical Composition

**Figure 1:**
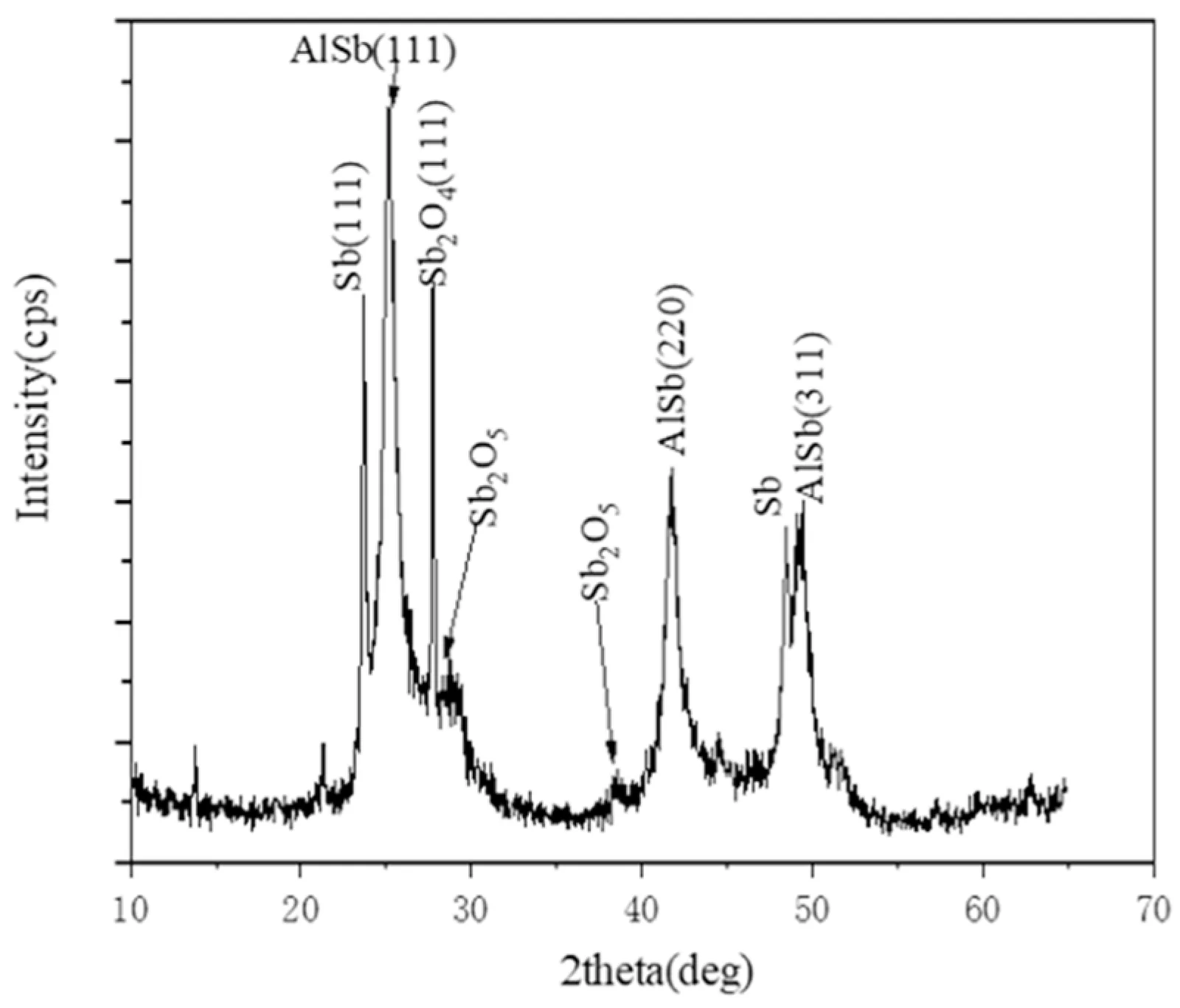
XRD pattern of AlSb thin film placed in air for 1 day.

To understand the deliquescence mechanism of AlSb thin films, the XRD of AlSb films was tested after being left to stand for 1 day, as shown in Figure 1. As can be seen from Figure 1, in addition to AlSb and Sb, Sb_2_O_4_ and Sb_2_O_5_ also appear in the thin film, and they preferentially orient along the Sb_2_O_4_ (111) crystal direction, indicating that there is a certain component in the atmosphere that greatly affects the stability of the AlSb film [20].

**Figure 2:**
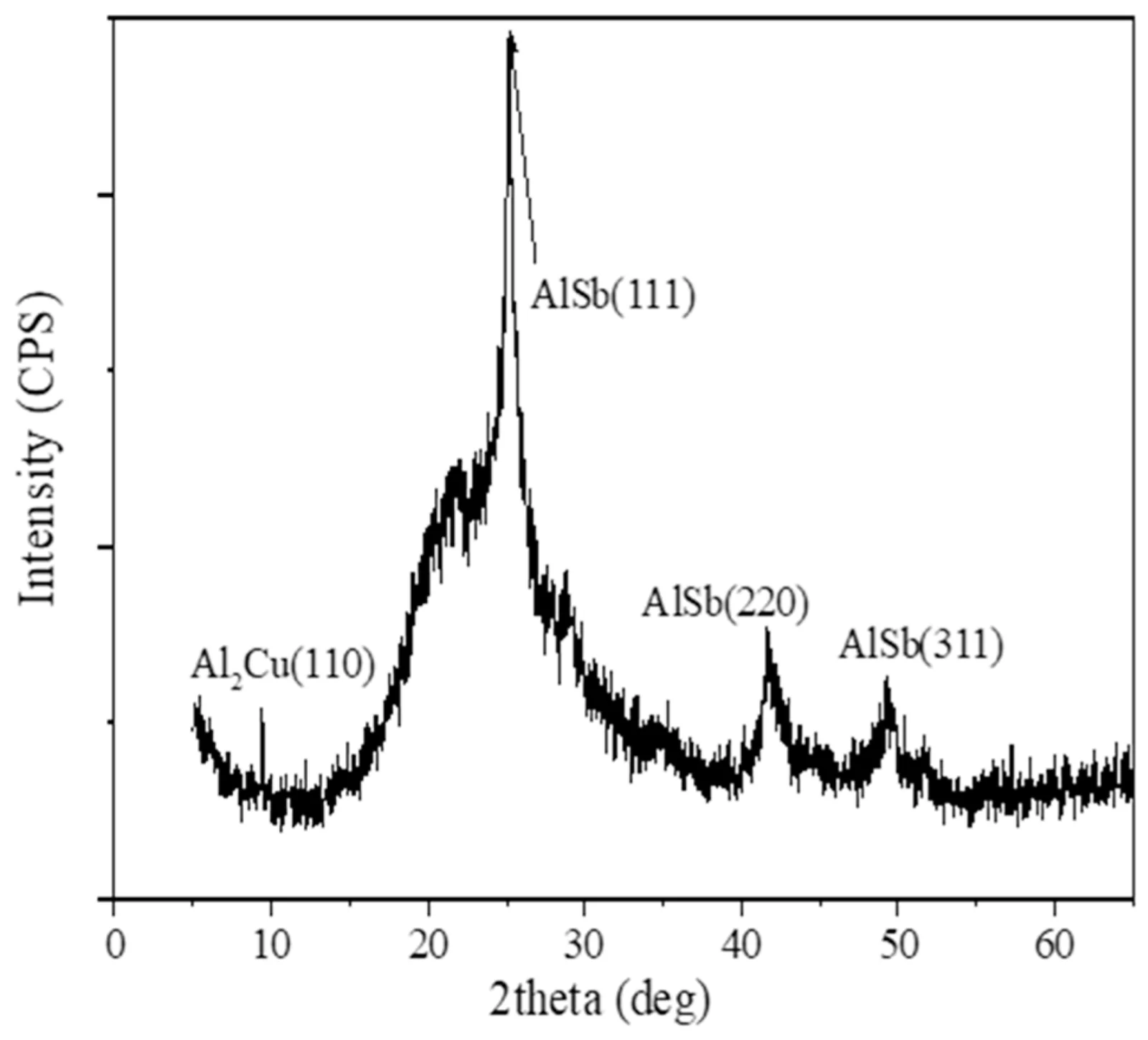
XRD pattern of AlSb:Cu thin film placed in air for 1 day.

The XRD pattern of an AlSb:Cu thin film with a Cu doping concentration of 6.25 atm% is shown in Figure 2 after being annealed at 400 °C for 1 h and then left to stand for 1 day. As evident from Figure 2, the curve exhibits peaks corresponding to AlSb (111), AlSb (220), and AlSb (311) crystal orientations, with preferential growth occurring in the AlSb (111) crystal orientation. The thin film structure is cubic. In addition, an Al_2_Cu (110) crystal orientation peak has appeared, which may be formed by the diffusion of the Cu protective film sputtered on the surface into the AlSb film layer after annealing, replacing the Sb atoms that exist in elemental form. The replaced Sb atoms are solid-solved into the AlSb lattice or Al_2_Cu lattice and exist in the form of a solid solution.

In order to research the process of AlSb thin films exposed to ambient air, elements Chemical states of the AlSb interface were analyzed.

**Figure 3:**
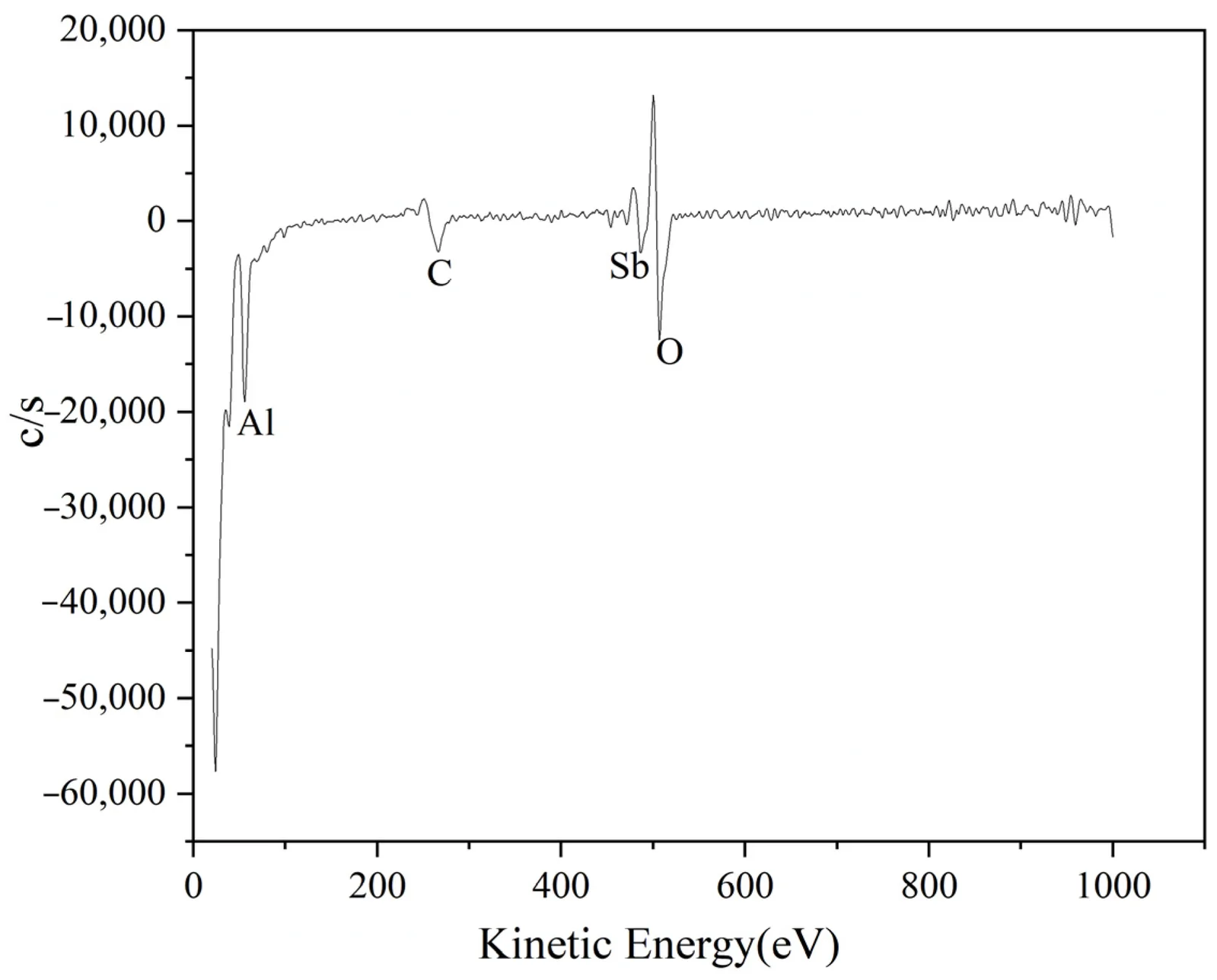
AES full spectrum of AlSb film.

AlSb film surface was stripped by Ar sputtering using a sputtering power of 4000 W, and the elements Chemical states of the interface were measured every 30 s using AES technology until the AlSb films were completely stripped. Figure 3 shows a typical AES full spectrum of AlSb films, from which it was clearly observed that the samples contained elements of Al, Sb, C, and O. The C element may come from CO and CO_2_ within the vacuum diffusion pump system during the deposition process. In addition to the residual gas in the vacuum chamber during the deposition process, element O may be due to the oxidation of AlSb films due to their contact with the atmosphere.

**Figure 4:**
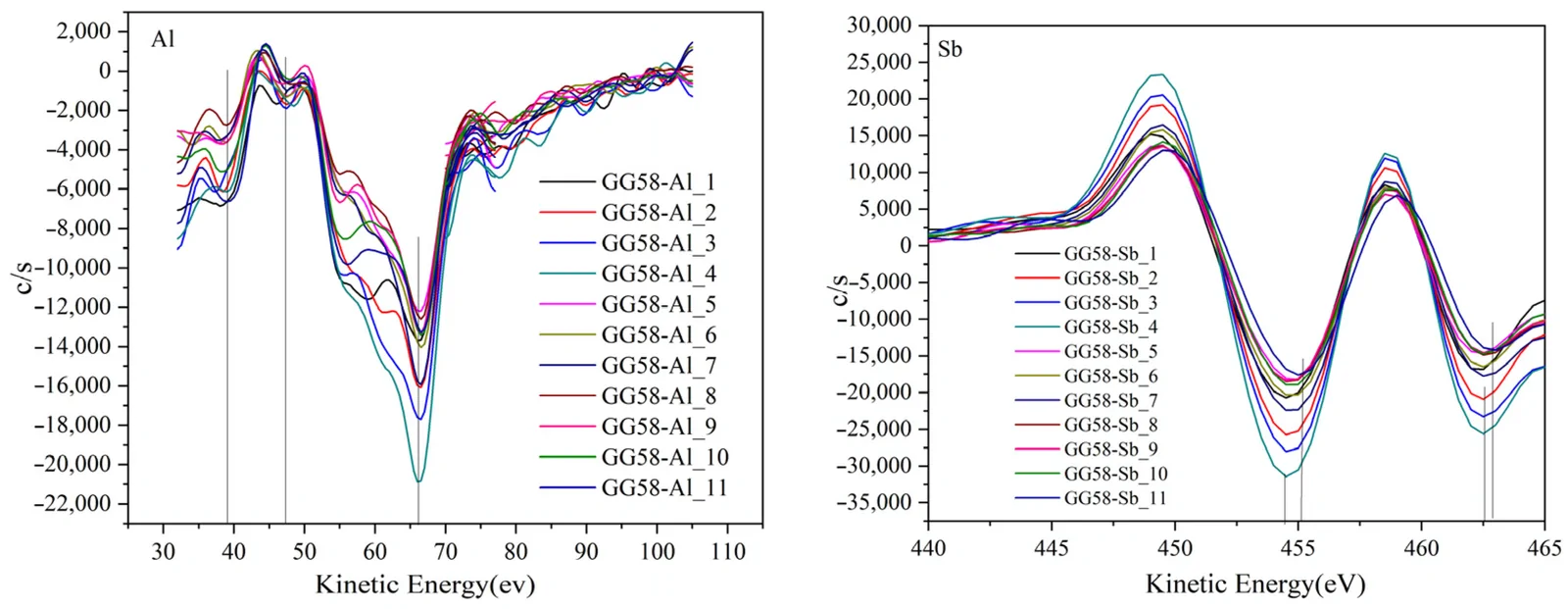
AES single spectrum diagram of Al and Sb elements in AlSb film.

The AES spectra of AlSb films are shown in Figure 4. As can be seen from Figure 4, there were two characteristic peaks of Al, located at 52 eV and 65 eV. The Auger peak at 66 eV may correspond to elemental Al solid-solubilized in the AlSb film, while the peak at 52 eV is characteristic of Al oxide. As the sputtering depth increased, the characteristic peak position of aluminum remained unchanged, indicating that the aluminum element had been completely oxidized. The kinetic energy of Sb was shifted to the right. The first peak was shifted from 454.6 eV to 455.2 eV (a shift of 0.6 eV), and the second peak was shifted from 462.5 eV to 463 eV (a shift of 0.5 eV). This may be caused by the continuous diffusion of O_2_, H_2_O, or CO_2_ molecules from the surface into the interior of AlSb, leading to the reconstruction of the electron cloud around the Sb atoms and resulting in minor fluctuations in their inner electron energy levels. Based on previous studies, the valence of the Sb element gradually changes from −3 to +5 from the interior to the surface [22]. Therefore, the antimony element in the interior of the film still exists in the form of an AlSb compound, while the electronic energy levels of the antimony element in the near-surface region undergo changes, leading to internal charge transfer of Sb atoms and resulting in valence changes [23]. Han et al. [24] and Tong et al. [25] reported oxygen-dependent indirect oxidation in the aqueous or water vapor region under aerobic conditions) and the generation of hydroxyl radicals (•OH) and other reactive oxygen species (ROS) may accelerate the dissolution or oxidation of Sb (III) near the surface. In 2026, Jun Shan reported that oxygen regulates the pathway and the location of Sb (III) oxidation rather than exerting a uniform promoting or suppressing effect [26].

**Figure 5:**
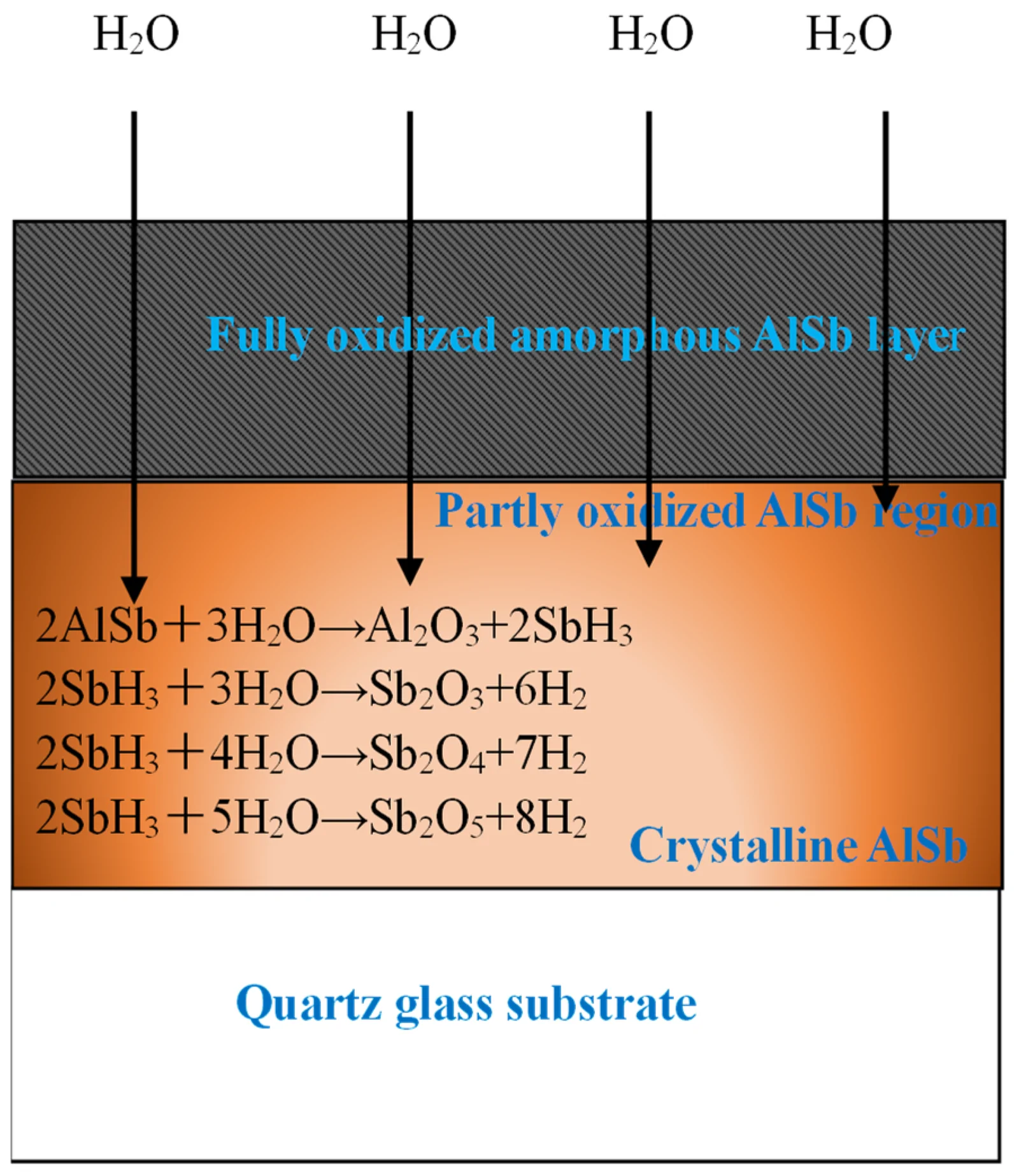
Adsorption oxidation process of AlSb thin film with H_2_O.

From the above analysis, the O element from H_2_O would gradually make Sb elements from the polycrystalline AlSb film in the atmosphere. H_2_O is adsorbed on the surface of the AlSb thin film in the atmosphere and undergoes a hydrolysis reaction to produce SbH_3_. The oxygen in the water gradually oxidizes SbH_3_ to antimony oxide, ultimately forming Sb_2_O_5_. The reaction process is shown in Figure 5.

AlSb thin films were easy to deliquesce and could not be preserved for a long time, which was the main reason that restricted their large-scale production. Our preliminary research has found that when water vapor comes into contact with AlSb thin films, sand holes will appear after the films are fully immersed in an atmospheric environment. Subsequently, water vapor reversely penetrates into the interior of the films, ultimately leading to structural spalling [22]. In order to study the mechanism of deliquescence, the adsorption energy of undoped AlSb thin films, as well as AlSb:Cu and AlSb:Zn films, was simulated by using the first-principles method from the molecular dynamics point of view using MS-2020 (Material Studio) software. The deliquescence mechanism of the AlSb film was revealed, and the change in interface properties was investigated.

## 3. Calculation and Theory Analysis

### 3.1. Crystal Cell Model of AlSb Thin Film and Doped Thin Film

**Figure 6:**
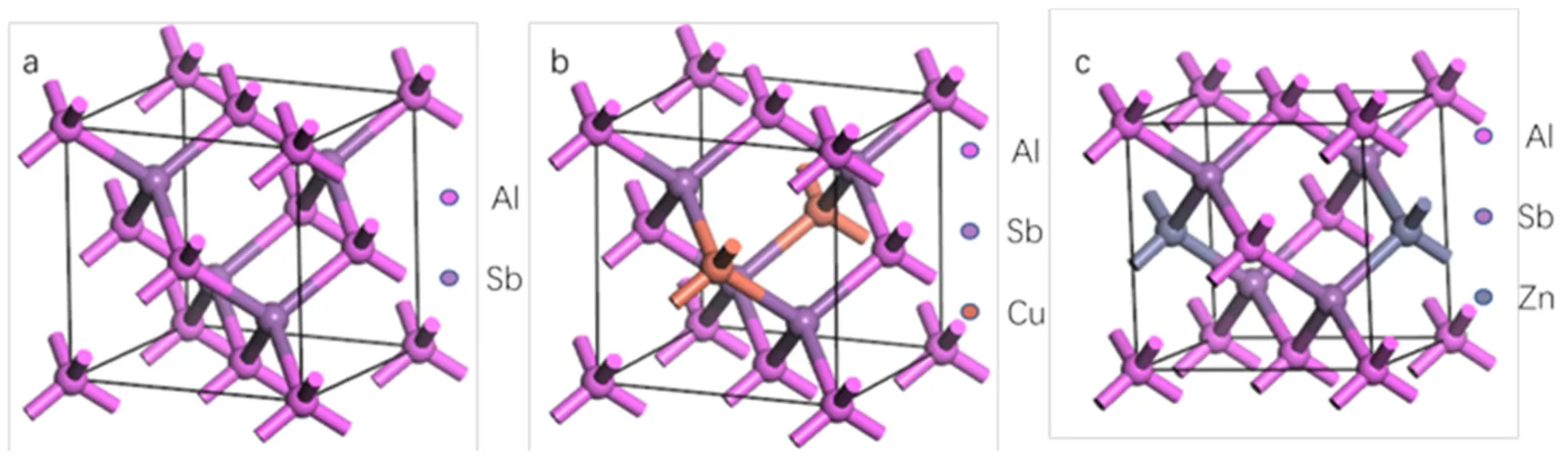
The structure diagram. (**a**) AlSb film. (**b**) AlSb·Cu film. (**c**) AlSb·Zn film.

The MS software was used to select the crystal cells of the basic AlSb polycrystalline thin film from its data and model it, as shown in Figure 6a. Cu and Zn atoms are mixed in the AlSb polycrystalline thin film, as shown in Figure 6b,c.

**Figure 7:**
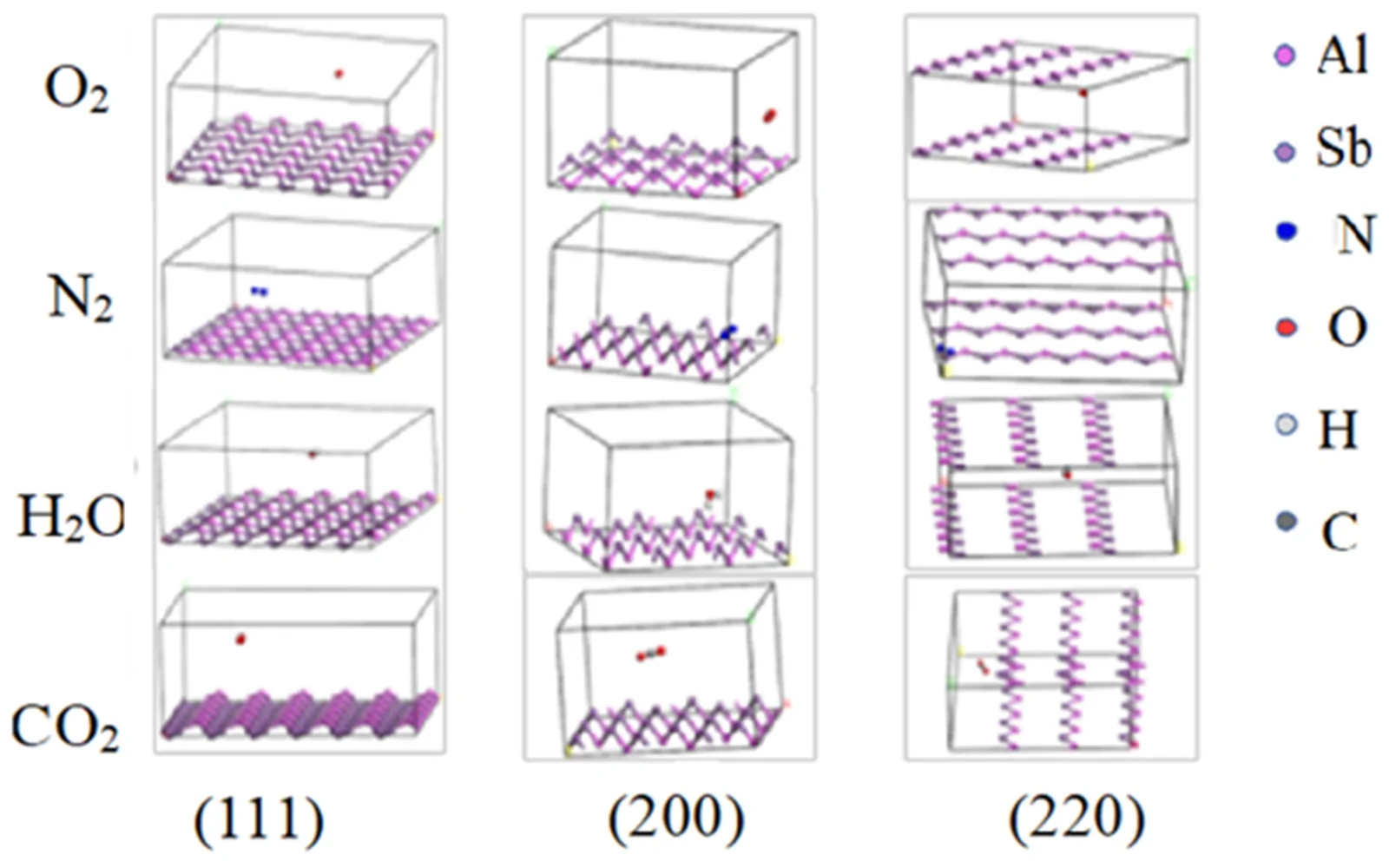
AlSb film flip each crystal plane combined with each molecule.

The most densely grown crystal planes of AlSb were selected by MS software: (111), (200), and (220), and the three crystal planes were sliced and expanded, and a vacuum layer was added to them to facilitate the subsequent addition of oxidizing molecules in the air, as shown in Figure 7.

**Figure 8:**
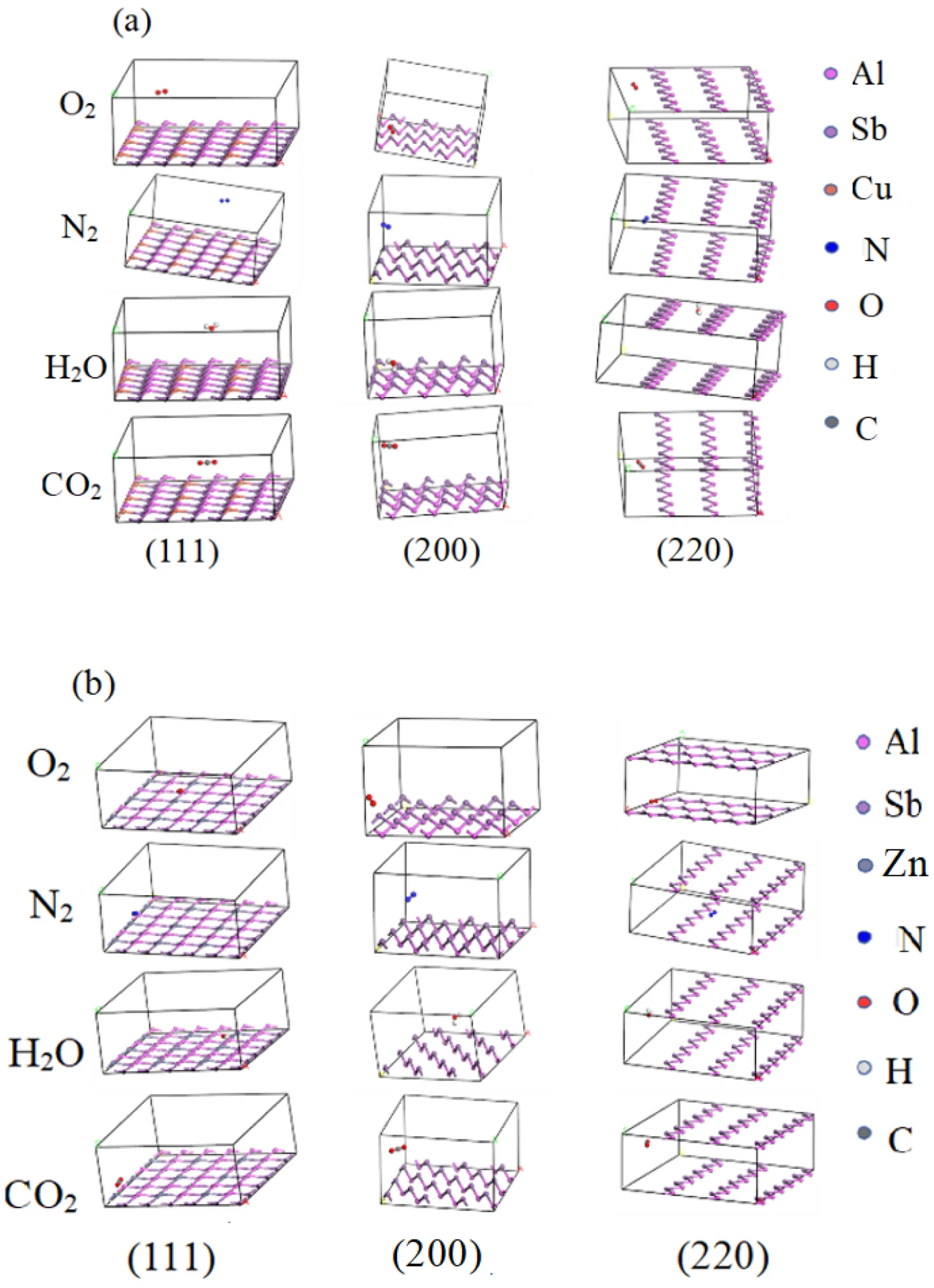
Schematic diagram of AlSb: Cu (**a**), AlSb:Zn (**b**) thin film combined with various molecules.

The oxidizing molecules (O_2_, CO_2_, N_2_, H_2_O) in the air were modeled separately, and the molecular models containing each oxidation were combined with the cross-sectional models of AlSb polycrystalline films, AlSb films doped with Cu atoms and Zn atoms, as shown in Figure 8. This combined model will be used in subsequent studies to investigate the interaction mechanism between the thin film surface and air molecules under different doping conditions.

### 3.2. Calculation and Analysis of Band Structure and Density of States

**Figure 9:**
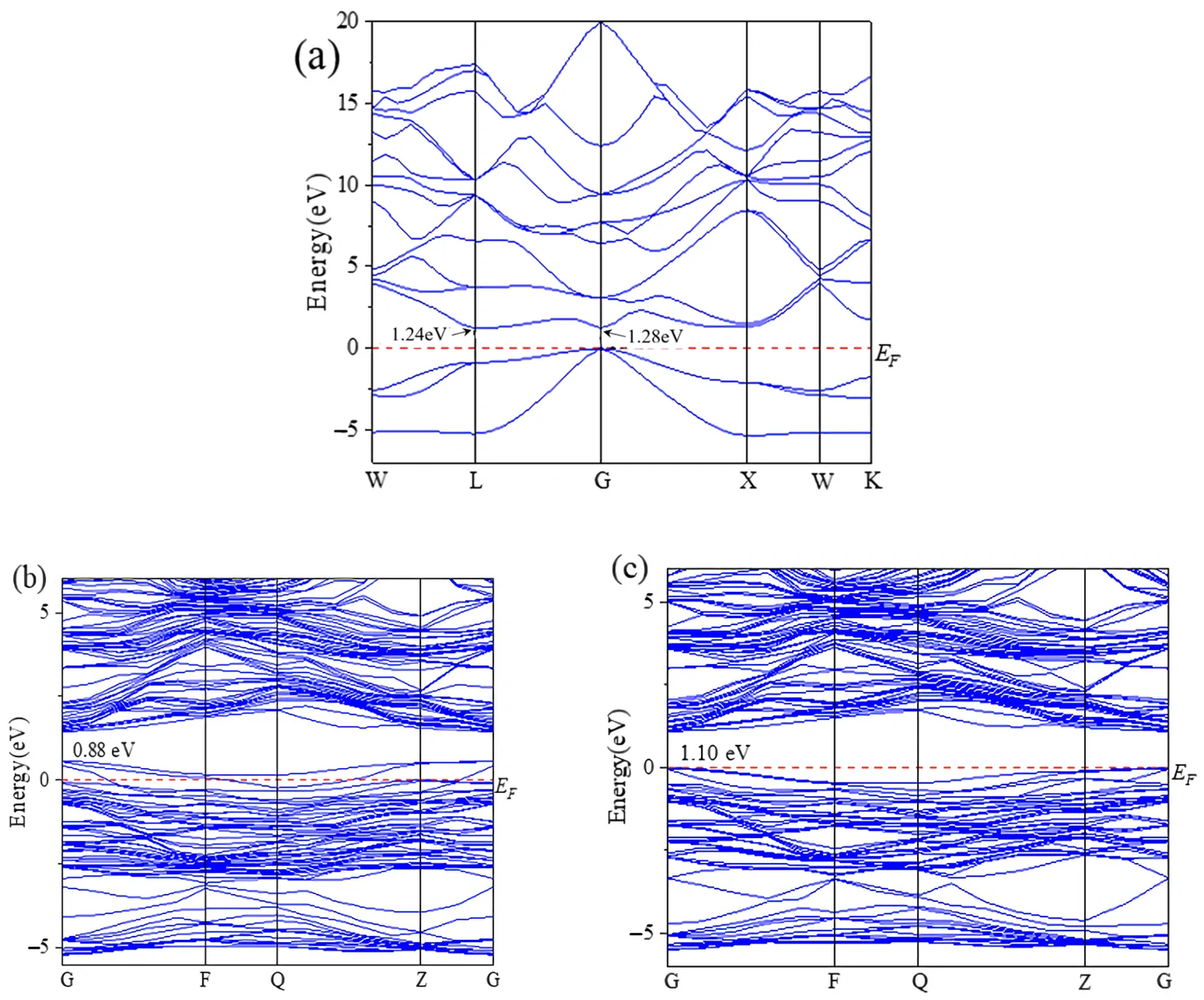
Band structure of AlSb thin film (**a**), AlSb:Cu (**b**) and AlSb:Zn (**c**) thin films.

Based on first principles and the generalized gradient approximation (GGA), the band structure and density of states of AlSb, which was doped with 6.25% atm Cu and 6.25% atm Zn AlSb were calculated using MS software. The band structures of AlSb thin film and AlSb: Cu (atm 6.25%) and AlSb: Zn (atm 6.25%) are shown in Figure 9.

From the band structure diagram in Figure 9a, it can be seen that the upper valence band ranges from −5.48 eV to 0 eV and the lower valence band from −10.92 eV to −8.92 eV, along with the conduction band, constitute the AlSb structure. The upper valence band exhibits splitting, forming three distinct subbands, while the conduction band consists of multiple subbands. Using first-principles calculations and the generalized gradient approximation (GGA), the minimum bandgap of the undoped zinc-blended-structured AlSb unit cell was calculated to be 1.124 eV, which is lower than the experimental value (Eg = 1.6 eV [2]). This discrepancy arises from the GGA approximation, as calculated Eg values tend to be lower than the actual theoretical values, a known limitation of the GGA method. The bottom of the conduction band and the top of the valence band are located at the L point and G point of the Brillouin zone, respectively, indicating that AlSb is an indirect bandgap semiconductor. After 6.25% Cu doping, the bandgap becomes 0.88 eV, and after 6.25% Zn doping, it becomes 1.10 eV. Thus, the bandgap width of the system decreases with the doping of Cu and Zn atoms, and the gap between the valence band and conduction band narrows. It can be seen that the top of the valence band in AlSb is primarily contributed by the p orbitals of Sb atoms, while the bottom of the conduction band is mainly contributed by the p orbitals of Al atoms.

**Figure 10:**
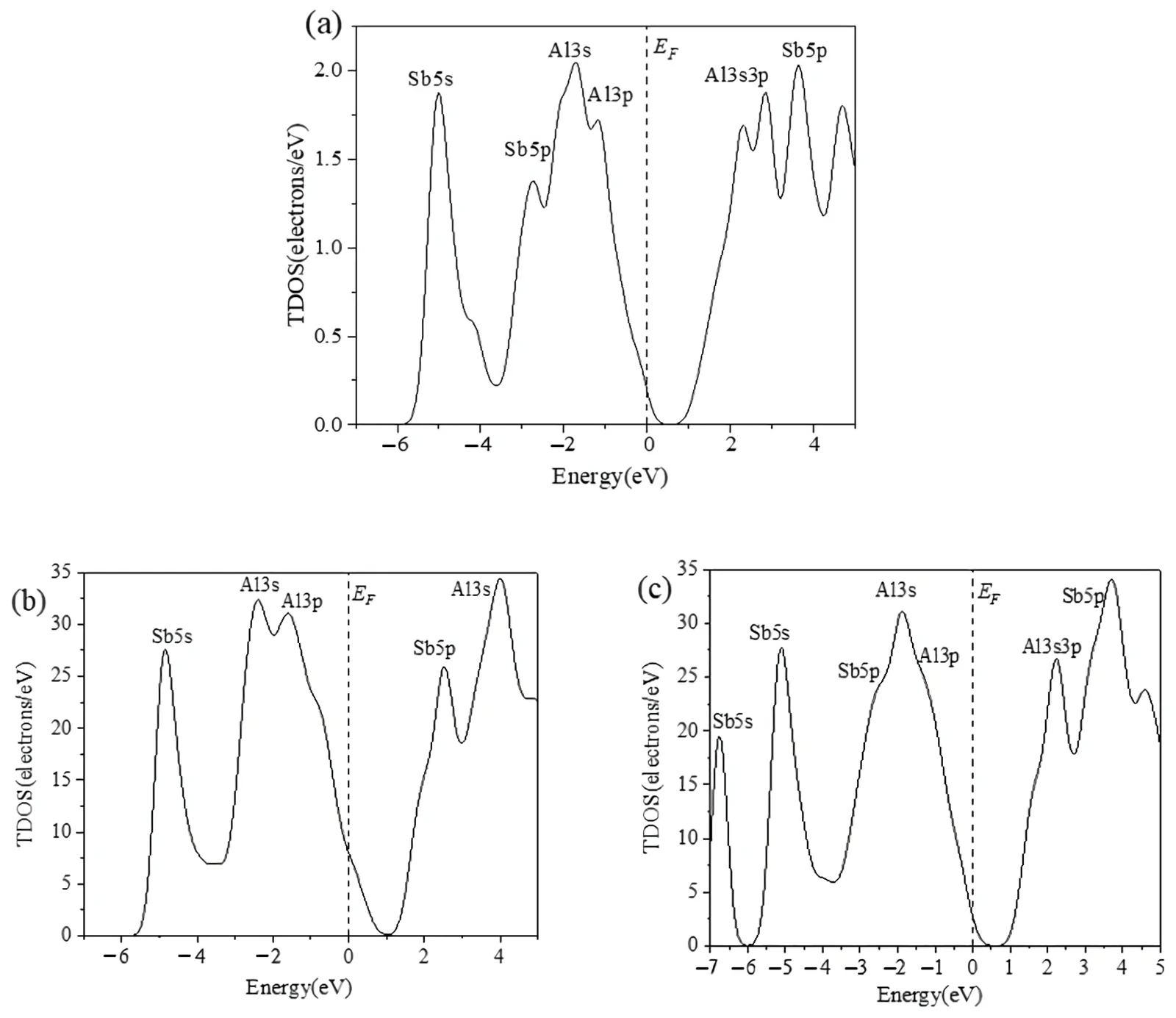
The density of states (DOS) plots for AlSb (**a**), AlSb:Cu (**b**), and AlSb:Zn (**c**).

Figure 10 shows the density of states (DOS) plots for AlSb thin films and AlSb:Cu (6.25% Cu atomic) and AlSb:Zn (6.25% Zn atomic). A comparative analysis of the band structure and DOS plots reveals that the upper valence band is formed by the Sb 5p and Al 3s3p states, while the lower valence band is primarily composed of the Sb 5s state. The Al 3p and Sb 5s5p states constitute the entire conduction band, with minor contributions from the Al 3s and Sb 5p states. The DOS of Cu-doped AlSb exhibits higher density near the top of the valence band compared to both pristine AlSb and Zn-doped AlSb, as this energy range corresponds to the 3D orbital states of the Cu dopant. Conversely, the Zn-doped AlSb shows an additional band near the bottom of the valence band due to the 3D states of the Zn dopant. After Cu and Zn doping, the energy levels exhibit partial splitting, likely caused by the substitution of Al atoms by impurity ions, which leads to 3D orbital splitting under the influence of Sb atomic bonding. Additionally, the formation of an Al-Cu alloy phase through bonding between Al and Cu atoms contributes to these structural changes.

### 3.3. Theoretical Foundations of Computing

The formula for the adsorption energy of an AlSb film after binding to oxidizing molecules is as follows Equation (1).

(1) $$∆E=\frac{E_{AlSb+air\;molecules}-E_{AlSb}-E_{air\;molecules}}{n}$$

∆E—Adsorption energy
$E_{AlSb+air\;molecules}$—The total energy of the AlSb film after binding to air molecules
$E_{AlSb}$—The energy of the system containing only the corresponding section of the AlSb film
$E_{air\;molecules}$—The system energy of air molecules
n—The number of molecules bound to each system

The calculation of the individual energy of each part is carried out by the calculation module included in the DMol^3^ module, and the calculation formula is shown in Equations (2)–(5).

(2) $${E=\Psi \;}^{\left(n\right)}(T+U+V)\Psi (n)$$

E—System energy
$\Psi^{\left(n\right)}$—Wave function
T—Kinetic energy term

(3) $$T(n)=-\frac{h^{2}}{2m}\sum_{i=1}^{N}\int d^{3}r{\Phi i}^{\left(r\right)}\nabla^{2}\Phi_{i}(r)$$

V—External potential energy term

(4) $$V\left(n\right)=\int V\left(r\right)n\left(r\right)dr$$

U—Interaction terms

(5) $$U\left(n\right)=\frac{e^{2}}{2}\int dr\int r^{\prime }\frac{n\left(r\right){n\left(r\right)}^{\prime }}{\left|r-r^{\prime }\right|}$$

All calculations were based on the DMol^3^ module in Materials Studio, utilizing density functional theory and numerical atomic orbital basis sets; the exchange-correlation functional was vdW-DF; a gradient optimization algorithm was employed for structural optimization to find the lowest energy. The total energy of the adsorption system, clean surface, and adsorbate was calculated through self-consistent field iteration, with a maximum of 500 steps. The energy convergence threshold was set at 0.002 Ha; the maximum force on atoms was converged to 0.002 Ha/Å. The SCF convergence precision was set at 1.0 × 10^−6^ Ha. The periodic system employed a surface slab model, with the k-point grid set as follows: the supercell was 5 × 5 × 1, and the vacuum layer was 5 × 5 × 10.

### 3.4. Data Analysis

#### 3.4.1. Binding Energy Analysis of AlSb Film with Air Molecules

After the geometry optimization of the density functional (DFT) quantum mechanical program DMol^3^ module of MS software, the energy changes in the main growth crystal planes of each molecule and each thin film in the air were calculated, as shown in Figure 9.

**Figure 11:**
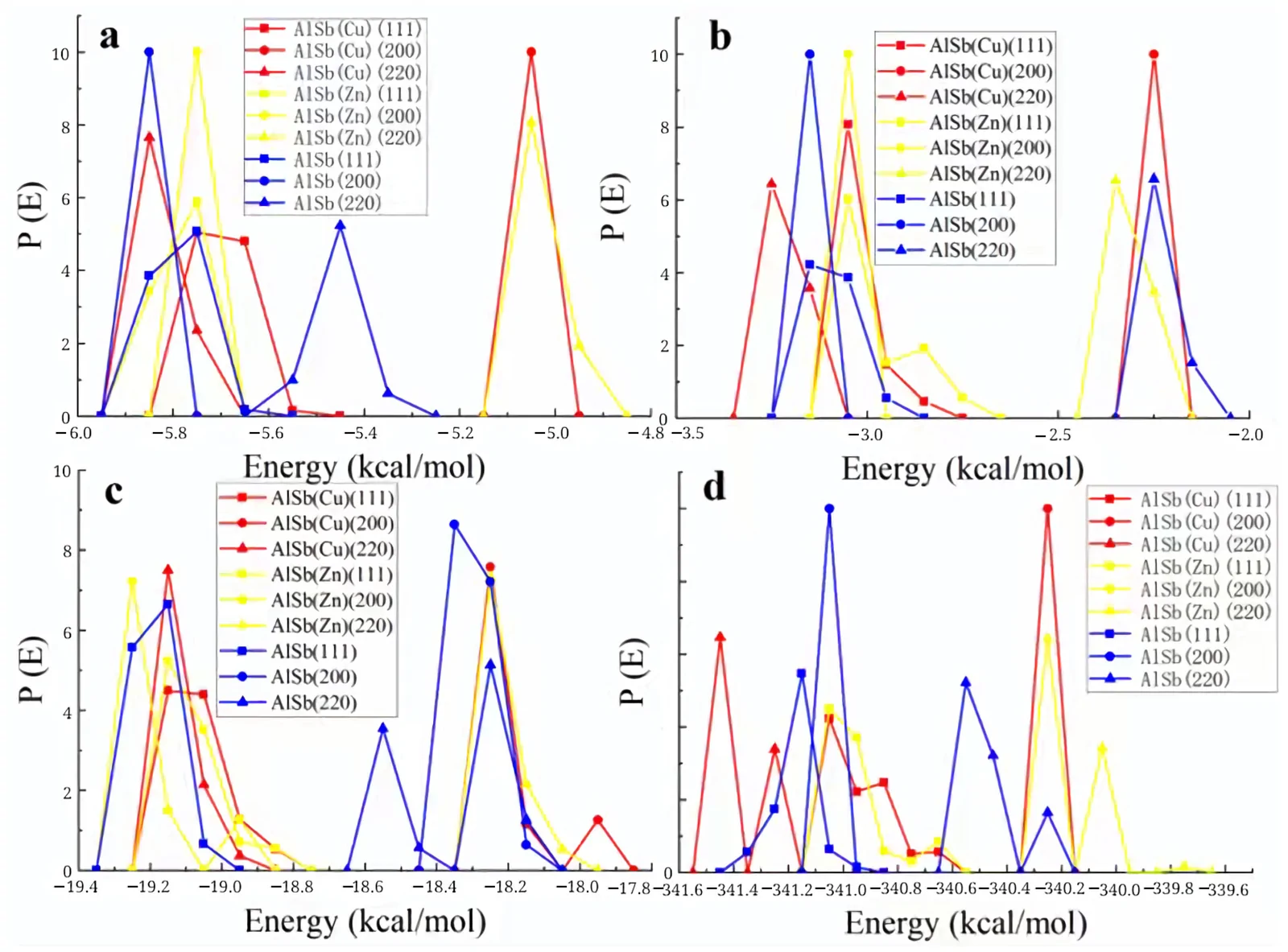
The change in energy of each molecule after binding to the model. (**a**) O_2_. (**b**) N_2_. (**c**) H_2_O. (**d**) CO_2_.

The absolute value of the energy of the O_2_ molecule was not significantly reduced relative to the (111) crystal of each film in Figure 11a. Compared with the (200) crystal planes of each film, the absolute energy reduction in O_2_ was greater than that of the films doped with Zn. For the crystal planes of each thin film (220), doping the Cu film can increase the absolute value of O_2_ energy, and doping Zn atoms can significantly reduce the absolute value of O_2_ energy. But none of the doped films significantly reduced the absolute energy value of N_2_ molecules for each film (111) crystal plane from Figure 9b. For the crystal planes of each thin film (200), the absolute value of N_2_ energy reduction was greater for the doped Cu film, while the absolute value of N_2_ molecular energy reduction was not obvious for the doped Zn film. For the crystal planes of each thin film (220), the doped film increased the absolute value of N_2_ molecular energy, and the doped Cu film increased the absolute value of N_2_ molecular energy more.

The absolute value of H_2_O molecular energy cannot be significantly reduced for each thin film (111) crystal plane from Figure 9c. For the crystal planes of each film (200), the absolute value of H_2_O molecular energy was only slightly reduced by Cu-doped films, while the absolute value of H_2_O molecular energy was increased by Zn-doped films. For the crystal planes of each thin film (220), the absolute value of H_2_O molecular energy increased by doping Cu film, while the absolute value of H_2_O molecular energy decreased by doping Zn thin film. However, from Figure 11d, for each film (111) crystal plane, the doped film can only slightly reduce the absolute value of CO_2_ molecular energy. For the crystal planes of each thin film (200), the doped Cu film significantly reduces the absolute value of CO_2_ molecular energy, while the doped Zn film was not useful to reduce the CO_2_ molecular energy. For the crystal planes of each thin film (220), the doped Zn film can reduce the absolute value of CO_2_ molecular energy, while the doped Cu film can increase the absolute value of CO_2_ molecular energy.

From the above analysis, it can be seen that the absolute reduction in the energy of various oxidizing molecules in the crystal face of the (200) AlSb film doped with Cu atoms was much better than that of the AlSb film doped with Zn atoms. The doped film had no effect on the absolute value of the energy of each molecule on the (111) crystal plane. The AlSb film doped with Cu atoms often had a counter-effect on the decrease in the absolute energy value of each molecule on the (220) crystal surface, while the AlSb film doped with Zn atoms only played a small disturbance effect on the absolute energy value of each molecule, and no obvious energy change occurred.

#### 3.4.2. Adsorption Energy Analysis of AlSb Films with Air Molecules

To further investigate the energy changes in various crystal faces in AlSb thin films and the mechanism of deliquescence of AlSb thin films, the adsorption energies of AlSb thin films with various molecules in the air were calculated, as shown in Table 1, Table 2 and Table 3.

As can be seen from Table 1, Table 2 and Table 3, whether it is the (111), (200), or (220) crystal plane of AlSb thin films, the adsorption energy of AlSb molecules toward H_2_O molecules in the air is the highest, releasing more energy and being in the most stable state. This explains the observation in previous experiments that the deliquescence process of AlSb is first combined with water vapor in the air, gradually penetrating from the film surface to the interior of the film, gradually forming sand holes on the substrate, and finally detaching from the substrate.

Therefore, to address the deliquescence issue of AlSb thin films and ensure their photoelectric performance in optoelectronic technology applications, doping of AlSb is necessary. Preliminary research has shown that doping AlSb thin films with Cu and Zn elements can maintain and enhance their photoelectric performance to some extent [8,22]. Therefore, the adsorption energies of various preferred crystal faces of AlSb:Cu and AlSb:Zn thin films toward various molecules in the air were calculated and analyzed. The results are presented in Table 4, Table 5, Table 6, Table 7, Table 8, Table 9 and Table 10.

From Table 1, Table 2, Table 3, Table 4, Table 5, Table 6, Table 7, Table 8, Table 9 and Table 10, it can be seen that the absolute value of the adsorption energy of H_2_O molecules after combining with the crystal plane was largest and most stable after combination among the same kind of AlSb films, resulting in the crystal plane being the easiest to combine with H_2_O molecules. The absolute value of adsorption energy was obviously higher before doping than after doping, indicating that the absolute value of adsorption energy was reduced after doping, and the binding state between the doped crystal plane and water molecules was more unstable than that before doping, and it was not easy to combine with water molecules. Among the same type of AlSb films, the crystal plane with the lowest adsorption energy and the easiest adsorption energy between the AlSb film doped with Cu atoms and H_2_O molecules was (200), the crystal plane with the lowest adsorption energy and the easiest adsorption plane between the AlSb film doped with Zn atoms and water molecules was (200), and the crystal plane with the lowest adsorption energy and the easiest adsorption between AlSb film and water molecules was (111).

## 4. Conclusions

The AlSb films were successfully prepared using direct-current (DC) magnetron co-sputtering technology. The results suggested that AlSb films were deliquescent and oxidized to oxides such as Sb_2_O_4_ and Sb_2_O_5_ after one day of contact with air. The chemical states of Al remained unchanged, and Sb in the films changed gradually from the surface to the interior. The crystal plane (111) of the AlSb film underwent an exothermic reaction with the molecules in the air, and the adsorption energy of H_2_O on the AlSb film was the highest, followed by that of O_2_, and the adsorption energy with N_2_ and CO_2_ was the lowest. After the AlSb films were doped with Cu and Zn atoms, the adsorption energy of the films with H_2_O and O_2_ molecules decreased, which could reduce the deliquescence oxidation of AlSb films and provide a theoretical basis for the application of AlSb films.

## Figures and Tables

**Table 1:** Adsorption energy of (111) crystal planes and molecules in AlSb films.

Model Type	The Energy of the System After Binding (kcal/mol·A^−2^)	AlSb Substrate System Energy (kcal/mol·A^−2^)	The Energy of a Single Oxidizing Molecular System (kcal/mol·A^−2^)	Adsorption Energy (kcal/mol·A^−2^)
O_2_	−3.1715642	−2.5956461	−5.7615462	−5.1856281
N_2_	−0.7651653	−2.4154845	−3.1431565	−4.7934757
H_2_O	−16.4981536	−2.8515463	−19.1715654	−5.5249581
CO_2_	−338.6511218	−2.5154613	−341.1215463	−4.9858858

**Table 2:** Adsorption energy of (200) crystal planes and molecules in AlSb films.

Model Type	The Energy of the System After Binding (kcal/mol·A^−2^)	AlSb Substrate System Energy (kcal/mol·A^−2^)	The Energy of a Single Oxidizing Molecular System (kcal/mol·A^−2^)	Adsorption Energy (kcal/mol·A^−2^)
O_2_	−3.6516471	−2.6623164	−5.8531614	−4.8638307
N_2_	−0.7816841	−2.5655434	−3.1131651	−4.8970244
H_2_O	−15.9231584	−2.9416546	−18.4341687	−5.4526649
CO_2_	−338.3134463	−2.4164194	−341.0615436	−5.1645167

**Table 3:** Adsorption energy of (220) crystal planes and molecules in AlSb films.

Model Type	The Energy of the System After Binding (kcal/mol·A^−2^)	AlSb Substrate System Energy (kcal/mol·A^−2^)	The Energy of a Single Oxidizing Molecular System (kcal/mol·A^−2^)	Adsorption Energy (kcal/mol·A^−2^)
O_2_	−3.7664184	−1.4949153	−5.4531463	−3.1816432
N_2_	−0.7926464	−1.6919455	−2.3648866	−3.2641857
H_2_O	−16.3343193	−1.7241531	−18.2141654	−3.6039992
CO_2_	−338.9261643	−1.5464597	−340.5314649	−3.1517603

**Table 4:** Adsorption energy of (111) crystal planes and molecules in AlSb:Cu films.

Model Type	The Energy of the System After Binding (kcal/mol·A^−2^)	AlSb Substrate System Energy (kcal/mol·A^−2^)	The Energy of a Single Oxidizing Molecular System (kcal/mol·A^−2^)	Adsorption Energy (kcal/mol·A^−2^)
O_2_	−3.67382744	−2.09825468	−5.77208212	−4.196509
N_2_	−0.76108285	−2.31175774	−3.07284059	−4.623515
H_2_O	−16.49867295	−2.59598658	−19.09465953	−5.191973
CO_2_	−338.8666416	−2.43736128	−341.3040028	−4.874723

**Table 5:** Adsorption energy of (200) crystal planes and molecules in AlSb:Cu films.

Model Type	The Energy of the System After Binding (kcal/mol·A^−2^)	AlSb Substrate System Energy (kcal/mol·A^−2^)	The Energy of a Single Oxidizing Molecular System (kcal/mol·A^−2^)	Adsorption Energy (kcal/mol·A^−2^)
O_2_	−3.67382744	−2.06595709	−5.73977961	−4.131914
N_2_	−0.76107491	−2.27581406	−3.03688896	−4.551628
H_2_O	−16.49855928	−2.70401231	−19.20257159	−5.408025
CO_2_	−338.8665405	−2.23180147	−341.098342	−4.463603

**Table 6:** Adsorption energy of (220) crystal planes and molecules in AlSb:Cu films.

Model Type	The Energy of the System After Binding (kcal/mol·A^−2^)	AlSb Substrate System Energy (kcal/mol·A^−2^)	The Energy of a Single Oxidizing Molecular System (kcal/mol·A^−2^)	Adsorption Energy (kcal/mol·A^−2^)
O_2_	−3.67383316	−1.40611701	−5.07995018	−2.812234
N_2_	−0.76108382	−1.5588861	−2.31996992	−3.117772
H_2_O	−16.49866751	−1.76478469	−18.26345219	−3.529569
CO_2_	−338.86663	−1.35357342	−340.2202034	−2.707147

**Table 7:** Adsorption energy of (111) crystal planes and molecules in AlSb:Zn films.

Model Type	The Energy of the System After Binding (kcal/mol·A^−2^)	AlSb Substrate System Energy (kcal/mol·A^−2^)	The Energy of a Single Oxidizing Molecular System (kcal/mol·A^−2^)	Adsorption Energy (kcal/mol·A^−2^)
O_2_	−3.67383093	−2.20703315	−5.88086408	−4.414066
N_2_	−0.76108168	−2.46412623	−3.2252079	−4.928252
H_2_O	−16.49866929	−2.65565753	−19.15432682	−5.311315
CO_2_	−338.8666329	−2.61448857	−341.4811215	−5.228977

**Table 8:** Adsorption energy of (200) crystal planes and molecules in AlSb:Zn films.

Model Type	The Energy of the System After Binding (kcal/mol·A^−2^)	AlSb Substrate System Energy (kcal/mol·A^−2^)	The Energy of a Single Oxidizing Molecular System (kcal/mol·A^−2^)	Adsorption Energy (kcal/mol·A^−2^)
O_2_	−3.67382296	−2.06101266	−5.73483563	−4.122025
N_2_	−0.76107454	−2.28054426	−3.04161879	−4.561089
H_2_O	−16.49855915	−2.70401244	−19.20257159	−5.408025
CO_2_	−338.8665405	−2.23180148	−341.098342	−4.463603

**Table 9:** Adsorption energy of (220) crystal planes and molecules in AlSb:Zn films.

Model Type	The Energy of the System After Binding (kcal/mol·A^−2^)	AlSb Substrate System Energy (kcal/mol·A^−2^)	The Energy of a Single Oxidizing Molecular System (kcal/mol·A^−2^)	Adsorption Energy (kcal/mol·A^−2^)
O_2_	−3.67383316	−1.40611701	−5.07995018	−2.812234
N_2_	−0.76108382	−1.5588861	−2.31996992	−3.117772
H_2_O	−16.49866746	−1.76478473	−18.26345219	−3.529569
CO_2_	−338.8666195	−1.35358332	−340.2202028	−2.707167

**Table 10:** Comparison of adsorption energies of various AlSb films.

Type of Film	(111) Adsorption Energy After Binding (kcal/mol·A^−2^)	(200) Adsorption Energy After Binding (kcal/mol·A^−2^)	(220) Adsorption Energy After Binding (kcal/mol·A^−2^)	Combine the Most Stable Crystal Planes
AlSb:Cu	−5.191973	−5.408025	−3.529569	(200)
AlSb:Zn	−5.311315	−5.408025	−3.529569	(200)
AlSb	−5.524958	−5.452664	−3.603999	(111)

## Data Availability

The original contributions presented in this study are included in the article. Further inquiries can be directed to the corresponding author.
